# Morphodynamic Foundations of Sumer

**DOI:** 10.1371/journal.pone.0329084

**Published:** 2025-08-20

**Authors:** Liviu Giosan, Reed Goodman

**Affiliations:** 1 Geology and Geophysics, Woods Hole Oceanographic Institution, Woods Hole, Massachusetts, United States of America; 2 STAR Institute, Babeș-Bolyai University, Cluj-Napoca, Romania; 3 Research Institute of the University of Bucharest (ICUB), Bucharest, Romania; 4 Baruch Institute of Coastal Ecology and Forest Science (BICEFS), Clemson University, Clemson, South Carolina, United States of America; 5 Museum of Archaeology & Anthropology, University of Pennsylvania, Philadelphia, Pennsylvania, United States of America; 6 Institute for the Study of the Ancient World, New York University, New York, New York, United States of America; Ariel University, ISRAEL

## Abstract

Economic mechanisms behind the emergence of ancient Sumer remain unavoidably speculative and should benefit from a better understanding of their environmental context. Abundance sustaining increased social complexity during the Uruk period (c. 6,000–5,200 y BP) has been traditionally ascribed to pastoralism, trade, and/or resource diversity. However, contemporary agricultural surpluses are hard to explain before adoption of large-scale irrigation systems. Here we use high-resolution satellite-based topography and paleoenvironmental proxies from a new drill core at Lagash/Tell Al Hiba, together with previous geological and archaeological data, to reconstruct the morphodynamic evolution of coastal Sumer. We propose that tidal irrigation offers a plausible jumpstarting mechanism for high-yield, diversified agriculture providing an impetus for urbanization. As access to sea was restricted by delta build-up and tides shifted with the advancing deltaic coast, intensified reliance on mercurial river regimes eventually led to the expansive fluvial irrigation network of Early Dynastic city-states. By positioning coastal morphodynamics as a pivotal factor in urbanization and political ecology, we underscore the intricate interconnections between naturally evolving systems and collective human agency.

## 1. Introduction

**Fig 1:**
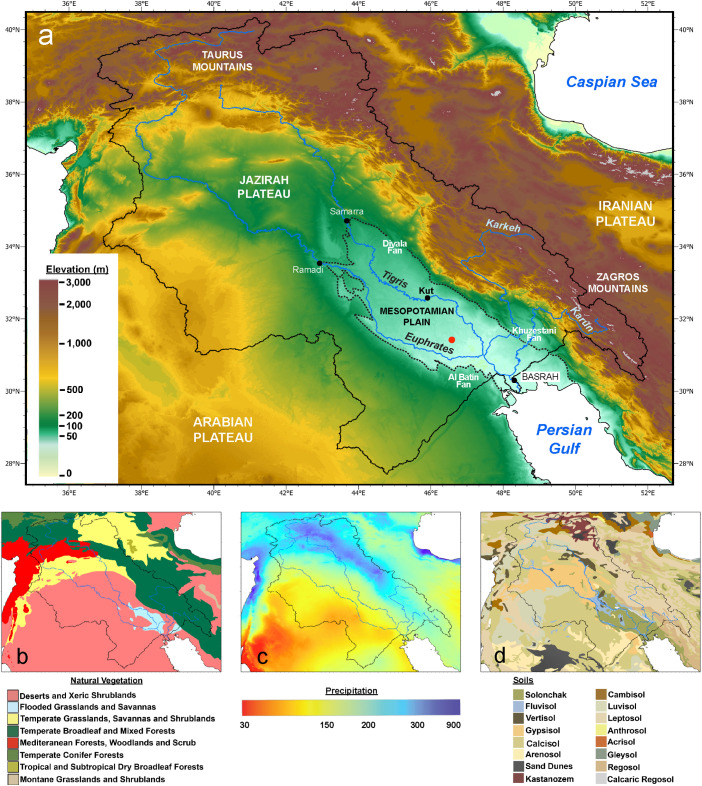
(a) Geography of Mesopotamian Plain (dashed black line) and its joint watershed (black line). Modern localities mentioned in text (black-filled circles respectively). Drill core location at Lagash in shown as red-filled circle; (b) Natural vegetation [7]; (c) Precipitation [8]; (d) Soils [9].

The earliest network of city-states [1], closely knit by shared cultural traditions and economic interests, emerged c. 5,000 years ago in southern Mesopotamia (Fig 1a). Collectively referred to as Sumer, this urban florescence was agrarian in nature, sustained by large-scale irrigation systems [2–4]. The urbanization of Sumer consolidated a process that started at least a millennium earlier, during the Uruk period (c. 6,000 BP–5,200 BP), following the long-lasting rural Ubaid culture. Synergistic increases in population, innovation, and occupational specialization at that time led to the appearance of state structures with complex economies, integrating an urban core with its rural periphery [5]. This “Sumerian takeoff” could not have happened in the harsh arid tropical zone between the Arabian and Iranian deserts without access to the perennial freshwater sources of the Euphrates and Tigris rivers. However, it remains uncertain when labor-intensive large-scale irrigation was widely adopted in the region and, prior to that, if and how this water abundance advantage translated into societal affluence.

Unlike ancient Egypt or the Indus Valley, flood recession irrigation was hindered in Mesopotamia by the long temporal lag between agricultural and fluvial cycles. Solving this “perplexing mismatch” [6] required complex engineering solutions to allow for irrigation during low river flow and flood protection during high flow. Cuneiform texts and radiocarbon-dated canals attest to such large-scale hydraulic works from c. 4,500 BP, but during the preceding proto-literate Uruk period, irrigation is only indirectly inferred [2,4]. Although pastoralism, trade, and resource diversity played important roles in the rise of Uruk, the origin of any agricultural surpluses that may have contributed to its unprecedented prosperity is uncertain. In this context, the agroecology of Sumer, which, in addition to river dynamics, was conditioned by the inland extent of the Persian (Arabian) Gulf waters and the configuration of its coastal zone, needs to be better resolved.

Here we provide a synoptic-scale reconstruction of coastal Sumer by combining existing geological and archaeological data with state-of-the-art satellite-acquired topography and paleo-environmental proxy records on a new drill core that we recovered on the lower Mesopotamian Plain at modern Tell al-Hiba/ancient Lagash (Fig 1a). The history of infilling at the head of the Persian Gulf started with a tidally-influenced Sumer delta lobe built with contributions from both Euphrates and Tigris rivers. Alongside a transversal fan-delta built by Karun and Karkeh rivers progressively blocked the head of the Gulf and ultimately merged with the Euphrates lobe into the emergent Shatt al-Arab delta. The remnant Mesopotamian Bay continued to be infilled by the Tigris with successive river-dominated lobes before joining the Shatt al-Arab. Based on morphodynamic considerations we argue that the mutual adjustment of river tides and coastal landforms at the head of the Gulf controlled the inception and evolution of Sumerian agriculture and represents an important key to understanding the cultural ecology of early urbanization and development of state institutions in Sumer.

## 2. Materials and methods

### 2.1. Geomorphology

**Fig 2:**
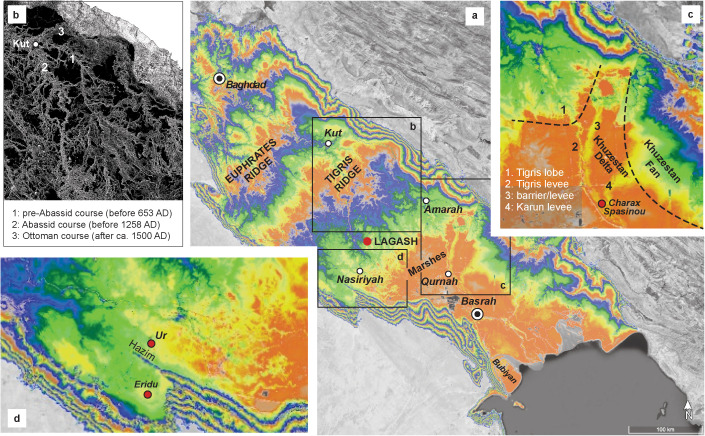
(a) Large-scale morphology of the Mesopotamian Plain based on Copernicus Sentinel data [10]. For altitudes, rainbow colors repeat every 10 m from 0 to 50 m in height; lands higher than 50 m are in gray; (b) avulsion node at Kut with historically-attested courses is shown in a gray-scale slope representation of the terrain model (higher slopes are lighter); (c) morphological elements of the confluence zone between Tigris and Khuzestan rivers; (d) the Eridu basin.

We used satellite-acquired topography data in combination with Google Earth to map the large-scale features of the Mesopotamian Plain. High-resolution (30-m) surface elevation data obtained from the Copernicus database [10] was used to construct a digital elevation model for the Mesopotamian Plain at 100-m resolution. To avoid misinterpreting the morphology due to post-Sumerian alterations of the Mesopotamian Plain via natural and anthropogenic processes, we only interpreted features that retain a clear, large-scale topographical expression: terraces and incised valleys, fluvial ridges, alluvial/fluvial fans, river levees, and coastal landscape elements (Fig 2).

### 2.2. Lithostratigraphy and paleoenvironment

Stratigraphy of the uppermost Mesopotamian Plain fill was investigated at Lagash (31° 25’ 16.716” N; 46° 25’ 9.156” E) in a drill core we recovered in 2022. Percussion drilling was employed to recover the sedimentary record to 25 m below surface; the preferred method of drilling used a corer with a PVC liner. In several instances, where loose sediments were encountered, the corer was replaced with a metal bailer with a core catcher. A 2 m deep trench was dug next to the coring location to expose the uppermost facies that was lost during the drilling process. For facies and paleoenvironment interpretation we reviewed information for boreholes available in previous publications on the Holocene stratigraphy and paleoenvironment of the Mesopotamian Plain [11–22].

**Fig 3:**
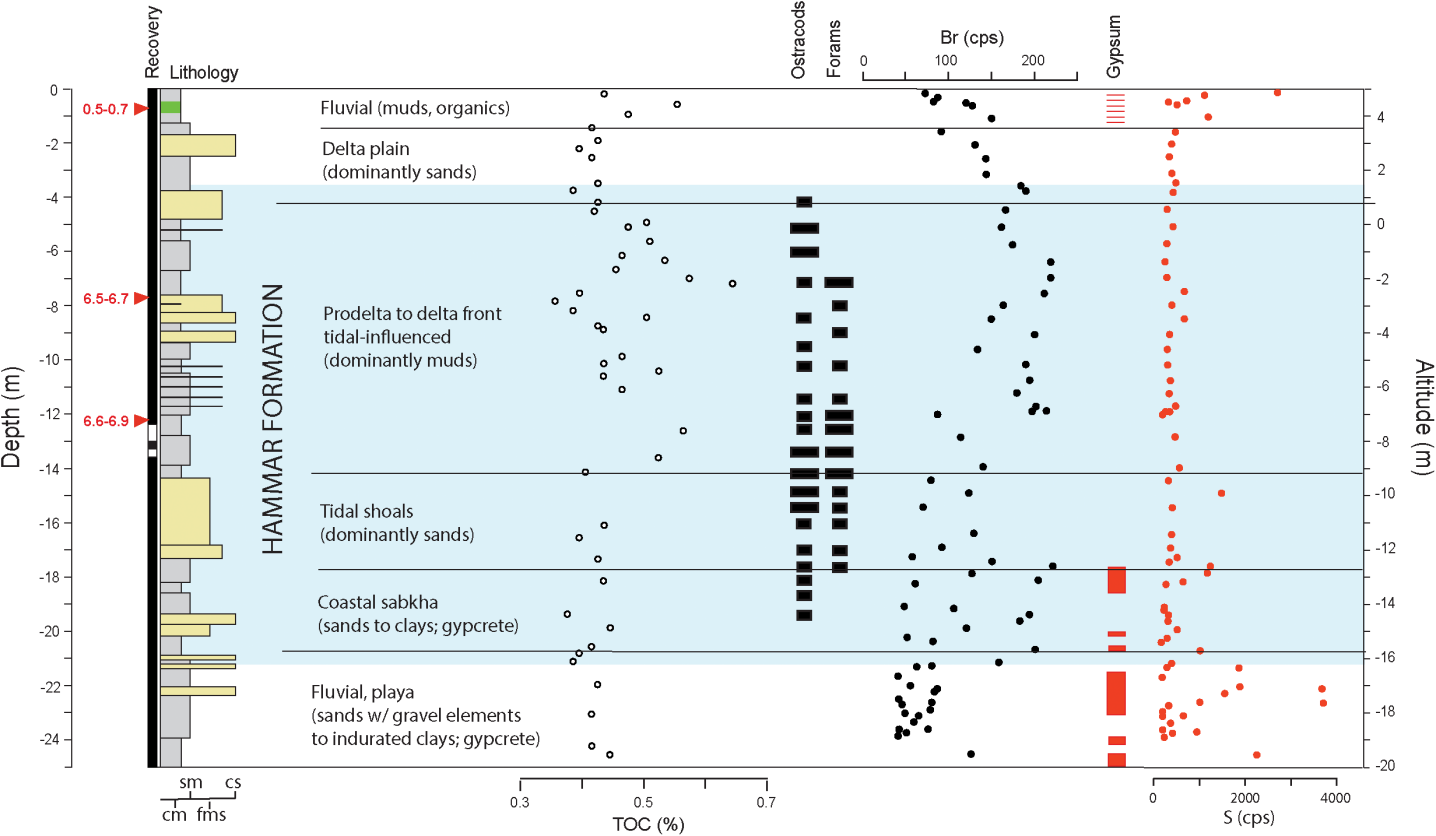
Depositional environments interpreted from litho-, bio-, and chemo-stratigraphy of Lagash drill core. In-situ radiocarbon dates (in thousands of years) are shown with red arrows. Paleoenvironmental proxies: total organic carbon (TOC); ostracods and foraminifers (present/abundant with short/long black bands respectively); bromine (Br); gypsum (red bands); and sulfur (S).

Based on lithofacies characteristics (i.e., lithology, textures, structures, bedding, biogenic and authigenic elements), we identified and logged fluvial, deltaic, and marine/tidal deposits (Fig 3). Chemostratigraphy was evaluated using Cox Itrax scanners at Woods Hole Oceanographic Institution and Lamont-Doherty Earth Observatory (S1 Table). Bromine and sulfur content were used to identify the presence of marine organic matter and gypsum, respectively [23,24]. Carbonate-free total organic carbon (TOC) was measured on 46 samples at the University of New Hampshire, using an Elementar Americas Pyrocube elemental analyzer (S2 Table). Prior to TOC analysis, inorganic carbon (IC) was dissolved using 6% sulfurous acid applied to weighed samples in amounts and steps optimized for carbonate-rich sediments [25].

Selected sediment samples were sieved and examined for microfossils, foraminifera, and ostracods to assess information on paleoenvironmental context and biostratigraphy through presence, abundance, and species composition. Foraminifera were used to infer intrusion of seawater leading to marine and/or brackish conditions [15], whereas ostracods served as indicators of standing water regardless of salinity. Separation and microscope study of microfauna was performed at Stony Brook University at a sample resolution of ~50 cm. Microfossil content was sparse and consisted of rare occurrences of ostracods (i.e., *Cyprideis*, *Limnocytheridae*, and *Darwinellidae*); benthic foraminifera were present (i.e., *Ammonia beccarii* and *Elphidium sp*.). Rare marine/brackish mollusks were also encountered (gastropod *Cerithidea cingulata* and bivalve *Saccostrea cucullata*) in the marine/estuarine sections, whereas freshwater species (gastropod *Melanoides tuberculata* and bivalve *Unio tigridis*) occurred in the fluvial intervals.

### 2.3. Chronology

**Fig 4:**
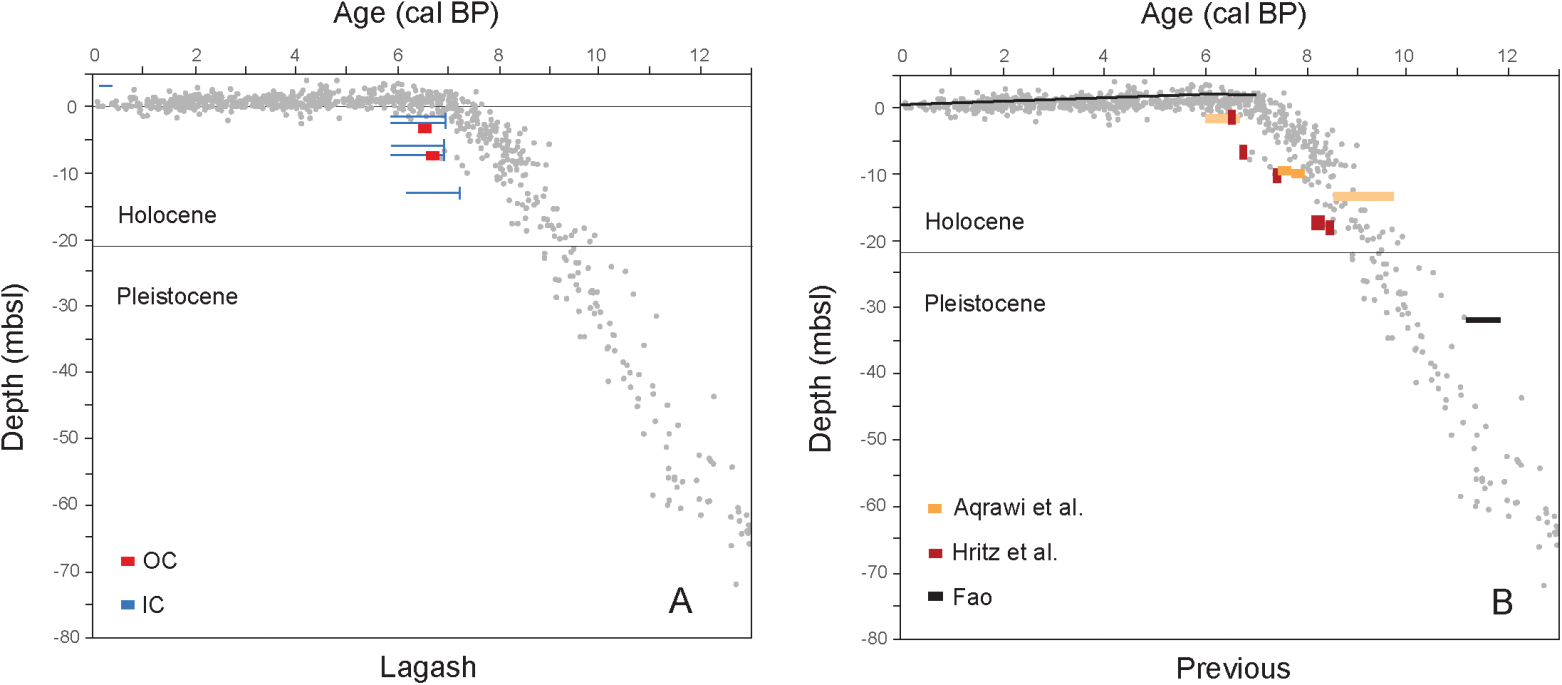
Calibrated radiocarbon dates from Iraqi cores [15,17,29,30] relative to the global sea level index points (from [31] shown as grey dots): (a) Lagash core (OC – organic carbon; IC – inorganic carbon); (b) previously reported and recalibrated. The highstand in the global dataset is confirmed by Persian Gulf data and the lowering trend since c. 6000 years ago [32] is indicated here by the black line in (b).

The chronology of the Lagash core was constructed based on accelerator mass spectrometry (AMS) radiocarbon dates on *in-situ* plant remains and carbonate bivalve/gastropod shells. (S3 Table; Figs 3 and 4). Dating sedimentation on the Mesopotamian Plain is difficult: the paucity of dateable organic material linked to harsh preservation conditions (i.e., aridity alternating with floods) is coupled with the mixing of extraneous materials that floods bring in. Bulk organic radiocarbon dates consist of a mixture of young in-situ production and old eroded organics generally rendering dates older [26]. On the other hand, inorganic carbon from carbonate shells is affected by variable fluvial reservoir ages [27], and, in the coastal zone, by the mixture of fluvial and marine conditions. The latter is also affected by fluctuating marine reservoir ages, typical for semi-enclosed, shallow conditions of the Persian Gulf [28].

All dates have been converted to years BP using OxCal 4.4 [33], the IntCal20: Northern Hemisphere atmospheric calibration dataset [34], and the Marine20: Modelled Ocean Average marine calibration dataset [35], which included ΔR = 180 ± 30 obtained from the Marine20 database. Given that the reservoir variability in the study region over the Holocene is unknown, a conservative approach was taken when estimating calendar ages of carbonate shells with a range presented as spanning both atmospheric and marine calibrations. The *in-situ* plant dates offer sedimentation ages and the tight overlap for the uncalibrated ages of the shells helps document the sedimentation rates. Radiocarbon dates from other publications [15,17,29,30] discussed herein (Fig 4b) were recalibrated.

## 3. Natural and cultural background

### 3.1. Environmental context

Sumerian settlements developed on the Mesopotamian Plain, also referred to as southern or lower Mesopotamia (Fig 1a). This vast, low-gradient alluvial plain was constructed through progressive sedimentary infilling of the Zagros Mountains’ foreland basin that formed during the convergence between Arabia and Eurasia. The natural landscape of the Mesopotamian Plain reflects long-term tectonics [36] as well as climate [37,38]. The Tigris-Euphrates river system, with its headwaters in the Taurus Mountains and flowing parallel to the Zagros, is the dominant sediment source for the region [39]. Short rivers draining the Zagros as well as streams activated on the Arabian Plateau during pluvials are other significant sediment sources.

Along their middle courses, the modern Euphrates and Tigris rivers are confined within terraced valleys, incised in the older sedimentary units of the Jazira Plateau or between the Jazira and geological formations of Arabia and Zagros (Fig 1a). As they emerge from their valleys downstream Ramadi and Samarra respectively, both rivers exhibit an avulsive behavior, sweeping across and depositing sediments on the plain [40]. Sediments from the Zagros rivers built alluvial/fluvial fans on the northeastern margin of the plain, with the Diyala fan and the Karun-Karkeh fan-delta complex in Khuzestan among the largest (Fig 1a). Similarly, on the Arabian side, alluvial fans such as al-Batin were apparently active during the Pleistocene.

During sea level lowstands, rivers extended into the Persian Gulf, the underfilled sector of the Zagros foreland, which was then a wind-modified, fluvio-deltaic landscape similar to the modern Mesopotamian Plain [36,37]. Natural river dynamics from Pleistocene into Holocene was controlled at first order by the baseline gradation to the Persian Gulf level that rose in the last transgression from c. −120 m at the Last Glacial Maximum (c. 20,000 years ago). A highstand of c. + 1–2 m was reached between c. 7,000–6,000 BP with the sea level decreasing since then to modern levels [32].

The near-surface stratigraphy of the Mesopotamian Plain is known from boreholes of the Geological Survey of Iraq [13]. Pleistocene fluvial sediments with frequent sand bodies are overlain by mud-dominated Holocene fluvio-lacustrine deposits [11–14]. The detailed pre-transgressive configuration of the Plain remains elusive, but the variability in Holocene cover between c. 10 and 20 m indicates a landscape with shallow incised valleys and slightly higher intervening mesas [13,41]. At the distal end of the Plain, the marine Hammar Formation, emplaced after the transgression [13,15,17], is primarily composed of the mud-rich submarine delta front deposits [13,15–19]. Subaerial delta plain and paludal sediments of the Mesopotamian Marshes representing the terminal infill stage at the head of the Gulf are typically more organic-rich with peats occurring sporadically [13,15,18]. Intercalated gypsum-rich deposits suggest a consistently arid environment on the Mesopotamian Plain through Pleistocene and Holocene [13,15].

At present, the mean annual temperature varies between 22°C in Bagdad and 24°C in Basrah, whereas precipitation rates are less than 250 cm per year across the entire Mesopotamian Plain, typical for a semi-desert. However, the natural vegetation consisting of flooded grasslands and savanna (Fig 1b) is explained by the water imported from upstream by the Tigris-Euphrates system (Fig 1c) and the dominance of fluvisols proper or salinized (Fig 1d) supports this relationship for the Holocene. Orographically-controlled precipitation in the Taurus and Zagros headwaters kept such large rivers perennial, which made the region less directly dependent on climate [42]. Consequently, the cultural ecology of lower Mesopotamia from initial settlement through urbanization is thought to have been contingent on river avulsions and sea level rise [36,40,43–45]. As we argue below, when sea-level rise rates began to decline during the latter half of the Ubaid period, the marine impact on coastal Sumer must have been primarily felt through modifications in waves and tides, modulated in turn by delta growth.

### 3.2. Brief Sumerian history

The Ubaid culture (c. 8,000–6,000 BP) is the earliest attested period of permanent habitation on the Mesopotamian Plain [46]. Associated settlements are known from low sedimentation sectors of the plain or deep soundings and excavations [47]. The long-lasting Ubaid was characterized by a scattered, non-hierarchical distribution of settlements that were relatively small. Agriculture, animal husbandry, fishing, and hunting supported populations with emergent craft specialization [48]. Irrigation was employed at a local scale [4] as indirectly inferred from archaeobotanical remains [49].

After c. 6,500 BP, Ubaid cultural elements appear in more remote settings along the Persian Gulf coasts as well as in northern Mesopotamia and western Iran [50]. Growing social stratification and the emergence of public temples [2] accompanied increases in the number of sites and their size (i.e., some larger than 10 ha). One of the largest settlements, located in a depression protected by higher ground, was the temple-town of Eridu [51]. This “first city” of later mythology was among the five “antediluvian cities” mentioned in the later-attested “Sumerian King List.” During the following Uruk period, settlement density increased, with more clustered and hierarchical site distributions organized around urban institutions [52]. Some sites reached several tens of hectares each. The site of Uruk itself (modern Tell al-Warka) underwent full-fledged urbanization reaching over 250 ha at its peak when it accommodated at least 25,000 people [53].

By 5,500 BP, Uruk-related settlements and/or cultural influences expanded northward into upper Mesopotamia and beyond, as well as eastward onto the Susiana Plateau in modern Khuzestan [50]. Even if details of precedence in the urbanization processes in the greater Near East are still being refined, the congruence of innovation, abundance, complexity, and extent, together with the emergence of state-like organizational structures, makes the Uruk period revolutionary and foundational [52]. New techniques were introduced or improved in agriculture, animal husbandry, fishing, manufacture and use of textiles, ceramics and metal, architecture, and art, among others. Most importantly, writing appeared toward the end of the period, primarily used in administrative tasks. Lexical and profession lists reveal a stratified society with complex economic ties across the region [54]. Although field-attested irrigation canals are yet to be dated to this period, these first texts document an exhaustive list of agricultural products that required relatively advanced subsistence techniques.

Cuneiform inscriptions become abundant after c. 4,600 BP, coincident with the world’s first “predominantly urbanized society” [2,55]. During the Early Dynastic period (4,900 BP–4,350 BP), city-state polities emerged integrating major urban centers by managed waterways. Dynastic rule replaced earlier power structures built around priest-kings and public assemblies [56]. Although societies became increasingly specialized and hierarchical, the population at large remained engaged in and dependent on agriculture. Control over labor was institutionalized and structured around large-scale hydraulic projects [57]. Important centers such as Ur, Uruk, Kish, Lagash, and Umma began to compete for control of water and irrigable land [58]. They developed alliances and ultimately entered conflicts. After an Akkadian imperial interlude, dynastic city-state revivals occurred, but the fall of the Third Dynasty of Ur (c. 4,000 BP) ended the primacy of Sumer as a dominant political network. In the two millennia of Sumerian history, the Uruk period stands out as particularly puzzling: what was the agro-economic basis of its demographic, social, and cultural effervescence that supported the emergence and refinement of state-like organizational structures?

## 4. Results and discussion

### 4.1. Sumer and the sea

During Holocene, until large-scale hydraulic works started to remodel southern Mesopotamia into a cultural landscape, fluvial landforms dominated in the upper part of the plain (Fig 2a). Through repeated avulsions (Fig 2b), the Tigris and Euphrates constructed stacked levees and splays coalescing into meander belts [40] and fluvial mega-ridges (Fig 2a). At the coast, fluvio-deltaic sediments infilled the pre-transgressive landscape (Fig 2c, d). The modern morphology (Fig 2a and 2d) retains a clear expression of the Khuzestan fan-delta, the successive river-dominated lobes constructed by the Tigris and the Shatt al-Arab tidal-influenced delta built by all rivers combined.

Early Sumerian urban settlements, including Uruk [38], developed close to the sea, but their precise proximity to the ancient coast remains unclear. Given the complex deltaic evolution in the region, the inland extent of the Persian Gulf in antiquity has been debated since archaeological interest in Mesopotamia began in the early 19th century [59]. However, the Hammar Formation [15,60] documents the marine transgression and can be used to roughly delineate the evolution of coast. At Lagash, the facies succession encountered in our core (Fig 3) conforms to and confirms the typical regional stratigraphy [13,15,19] with marine sediments sandwiched between fluvial deposits [61].

Starting from the surface floodplain/delta plain muds and sands continue down-core into the marine muds and fine sands of the delta front with occasional, intercalated flood/storm coarser layers (Fig 3). Radiocarbon-dates show that the delta lobe advanced rapidly over submerged tidal shoals near Lagash into a basin less than 10 m deep between c. 7,000 and 6,000 years ago, during the sea-level highstand (Fig 4). The underlying organic matter-poor sediments consist of tidal sands, similar to modern shoals at the mouth of Shatt al-Arab. They stand above intertidal/supratidal coastal sabkha muds that corresponds to the first transgressive sediments in the region. Typical Pleistocene fluvial indurated red clays to structureless fine to medium sands with gravel elements occur below the transgressive deposits. Evaporative conditions with increased sulfur and visually detectable gypsum are encountered in Pleistocene deposits, the early transgressive sabkhas and, later, on the recent floodplain.

### 4.2. The Mesopotamian delta complex

**Fig 5:**
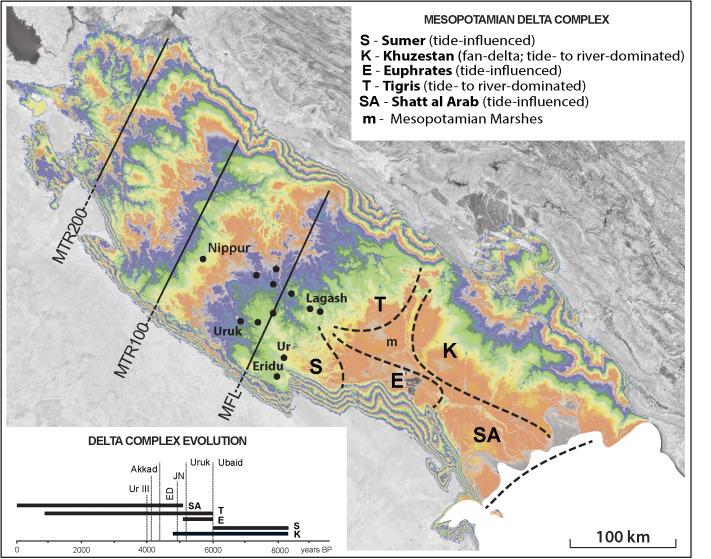
Evolution of the Mesopotamian delta complex composed of the axial-oriented Sumer lobe (S), Euphrates (E) and Tigris (T) lobes and Shatt al-Arab (SA) as well as the transversal Khuzestan fan-delta (K). Sumerian cities larger than 100 ha are shown with inferred maximum flooding limit (MFL). Upstream from the MFL, maximum tidal reaches are shown as MTR200 and MTR100 when the backwater zone in is assumed at 200 km (e.g., similar to Shatt al-Arab) and 100 km, respectively. The upper inset describes the lobe morphology, and the lower inset shows estimated time spans for delta lobes relative to cultural phases in Sumer.

Previous geological data [15,62–64] suggest that a tidally influenced delta lobe first developed along the southwestern edge of the Mesopotamian Plain before extending laterally toward our site at Lagash. Mineralogy [63] indicates that this first Sumer lobe (Fig 5) was built jointly by the Euphrates and Tigris. As the marine transgression probably did not reach as far north as Uruk [38], it appears that this joint river provided enough sediment for the delta to keep pace with the sea-level rise. The absence of Ubaid and Sumerian settlements along the lower modern course of the Tigris [41,46,62] agrees with subsurface geology [13] indicating that the sea-level transgression reached deeper inland in that region. Whether the Tigris split from the Euphrates as the delta plain emerged at Lagash remains an open question [61]. However, the persistence of a Tigris avulsion point near Kut (Fig 2b) supports a delayed deltaic infilling of the eastern side of the transgressive basin. Therefore, the maximum flooding limit (MFL), which roughly delineates the most inland transgressive coast, extended transversally across the Mesopotamian Plain from near Nasiriyah to below Kut (Fig 5).

Further along on the northeastern edge of the Mesopotamian basin, lateral to its axis, the Khuzestani rivers initially built a composite fluvial fan (Fig 5). After infilling their late Pleistocene incised valleys [65–67], the fan morphed into a delta by 5,000 BP. The rapid shoaling of the remaining access route to the Gulf [30], in the future Shatt al-Arab delta sector, occurred between 7,000 and 6,000 BP (Fig 4), probably fed by both axial and transversal sediments sources. It remains unresolved if the Tigris already split or still fed the Euphrates lobe when the latter joined the Khuzestan delta. However, this choking process of the Gulf’s head isolated behind a vast embayment, the Mesopotamian Bay, later to be infilled mainly by river-dominated deltaic lobes of the Tigris (Fig 5) and transformed into the Mesopotamian Marshes. The rapid and extensive reorganization of the head of the Persian Gulf, from open seacoast to a blocked bay, was contemporaneous with the similarly drastic transition from a rural culture toward a fully urban society (Fig 5). Below we argue that this dramatic landscape transformation regulated the type of agriculture practiced in coastal Sumer, which in turn favored urbanization and the emergence of the State.

### 4.3. Regional morphodynamics of coastal Sumer

In contrast to the better-known fluvial sector of the Mesopotamian Plain, the morphodynamics of coastal Mesopotamia remains a critical gap in understanding habitability and human settlement patterns in early Sumer. Elements responsible for the heterogeneous coastal palimpsest, such as the role of rivers as delta builders and the sediment trapping in the embayment behind the Khuzestani delta, were recognized early by investigators [41,44,45,59]. Focus on sea-level change, broadly understood, has shaped the scholarly discourse on coastal Sumer [20, 37, 41, 68] and a potential role for tides was proposed [6,41,69]. The drastic metamorphosis from open tidal coast to a blocked bay, gradually infilled by delta lobes, together with its potential impacts on agriculture and settlement, however, have not.

The morphology of tidally influenced deltas is quite distinctive and stable [70]: seaward-widening distributary channels accommodate augmented tide-modified fluvial discharges and self-organize to uniformly redistribute tides across the entire delta system. In the semi-diurnal mesotidal conditions of the Gulf (range ≥3 m), the modern Shatt al-Arab (Fig 5) exhibits such a tide-influenced morphology, typical for a narrow lobe pinched between the higher grounds of the Khuzestan fan-delta and al-Batin. This provides a suitable analog for the Sumer lobe that probably built initially along an incised valley connected downstream to similar valleys of the Khuzestani rivers [65–67]. Evidence from Ur [71] and Uruk [38] suggests that transgressive freshwater marshes were already established by c. 7,000 BP at these locations (Fig 5).

Today’s Shatt al-Arab also provides a hydraulic analog for tidally affected deltaic channels of the Sumer lobe. Typically for large rivers, dense marine waters enter the mouth as a salt wedge hugging the bottom; the wedge extends upstream on much shorter distances than the tidal reach of the backwater at the surface [72,73]. In natural conditions before upstream damming, the salt wedge of the Shatt al-Arab penetrated several tens of km, whereas tides raised the river levels up to 200 km inland [72]. Taking advantage of this hydraulic setting, tidal irrigation has been documented for large areas near medieval Basrah, probably feeding date groves and cereal fields [74]. As late as the 20^th^ century, tides irrigated some of the most productive date-palm farms in the world near the Shatt al-Arab [75] extending on a strip ~1–3 km wide over a distance >100 km along the river. In the shade of date groves, maintaining cooler and wetter microclimates, other fruits, legumes, and vegetables were cultivated. This agro-ecological practice was also central in Sumer [76,77] where dates had been a staple since the Ubaid period [76].

We argue that suitable conditions for tidal irrigation were ubiquitous in coastal Sumer. From the maximum flooding limit (MFL) at the sea level highstand, the maximum freshwater tidal reach (MTR) extended deep inland on Mesopotamian Plain (Fig 5). As the deltaic lobes later advanced, the maximum tidal reach correspondingly retreated toward the modern Gulf coast. If we use the tidal penetration on the modern Shatt al-Arab as a guide (i.e., 200 km), tides at maximum transgression could have been felt on river channels along most of the length of the Mesopotamian Plain (Fig 5). Even when assuming a much shorter tidal reach (i.e., 100 km) to account for steeper gradients, tides probably reached as far as Nippur (Fig. 5). Therefore, when the head of the Gulf was still open, favorable conditions for tidal irrigation must have occurred along the lower flood- and delta-plains of the Tigris and Euphrates, latitudinally conterminous with early communities like Eridu, Ur and Uruk.

In an estuarine setting such as the Mesopotamian Bay, the tidal range also dampens when its entrance becomes laterally restricted [78] so a significant decline in tides must have occurred between 6,000 and 5,000 BP, as the inlet to the Gulf shallowed and narrowed [30,65]. This transition was completed before 5,000 y BP as the tidal influence waned on the Khuzestani delta [67]. As early as c. 4,000 BP, the Bubiyan deltaic island (Fig 2) was fully emergent at the current coast indicating that the Shatt al-Arab delta lobe was in place [79]. Later delta lobes of the Tigris shifted to being fully river-dominated (Figs 2 and 5) in agreement the Mesopotamian Bay’s transition to microtidal conditions typical for the Mesopotamian Marshes [75]. To sum up, the deltaic infilling of the Mesopotamian Bay by Tigris and Euphrates and the constriction imposed by the Khuzestani transversal delta expansion were decisive in controlling tidal hydraulics at the head of the Gulf. We propose that, in turn, the change in tides restructured the agricultural and thus cultural ecology of the region during the transition from the pre-urban Ubaid culture into fully urbanized Sumer.

### 4.4. From tidal agriculture to large-scale irrigation

Taking morphodynamics into account, we can better understand the sequence in the emergence of cities as well as the time when society took on the role of landscape engineers at regional scale. The maximum extension of freshwater tidal reach upstream delineates an extensive zone, encompassing most of Sumer (Fig 5), where tidewater farming was feasible beyond the annual fluvial cycle previously invoked for flood recession agriculture [e.g., 46]. Herein also lies a potential answer to the early irrigation paradox: agriculture that does not require sophisticated large-scale canal networks and flood protection.

The tidal freshwater river zone was a particularly advantageous ecological niche for early experiments with agriculture as tides would have promoted channel stability that “allowed greater flexibility and predictability in the timing of cultivation” [69]. Tidal irrigation mechanics are steady, straightforward, and benign: the tidal cycle is not unpredictably destructive, while it functions naturally to irrigate the low-lying terrains beyond the river levee, propelling river water through human-made surficial canals extending laterally from the natural river channel. During the flood tide, overbank flow can be tapped to increase or decrease the amount of water flowing through such canals onto cultivated lands. With the onset of the ebb tide, the river levels drop, groundwater levels decrease, and the water returns to the rivers from the fields, flushing salts that may develop under high evaporation rates.

**Fig 6:**
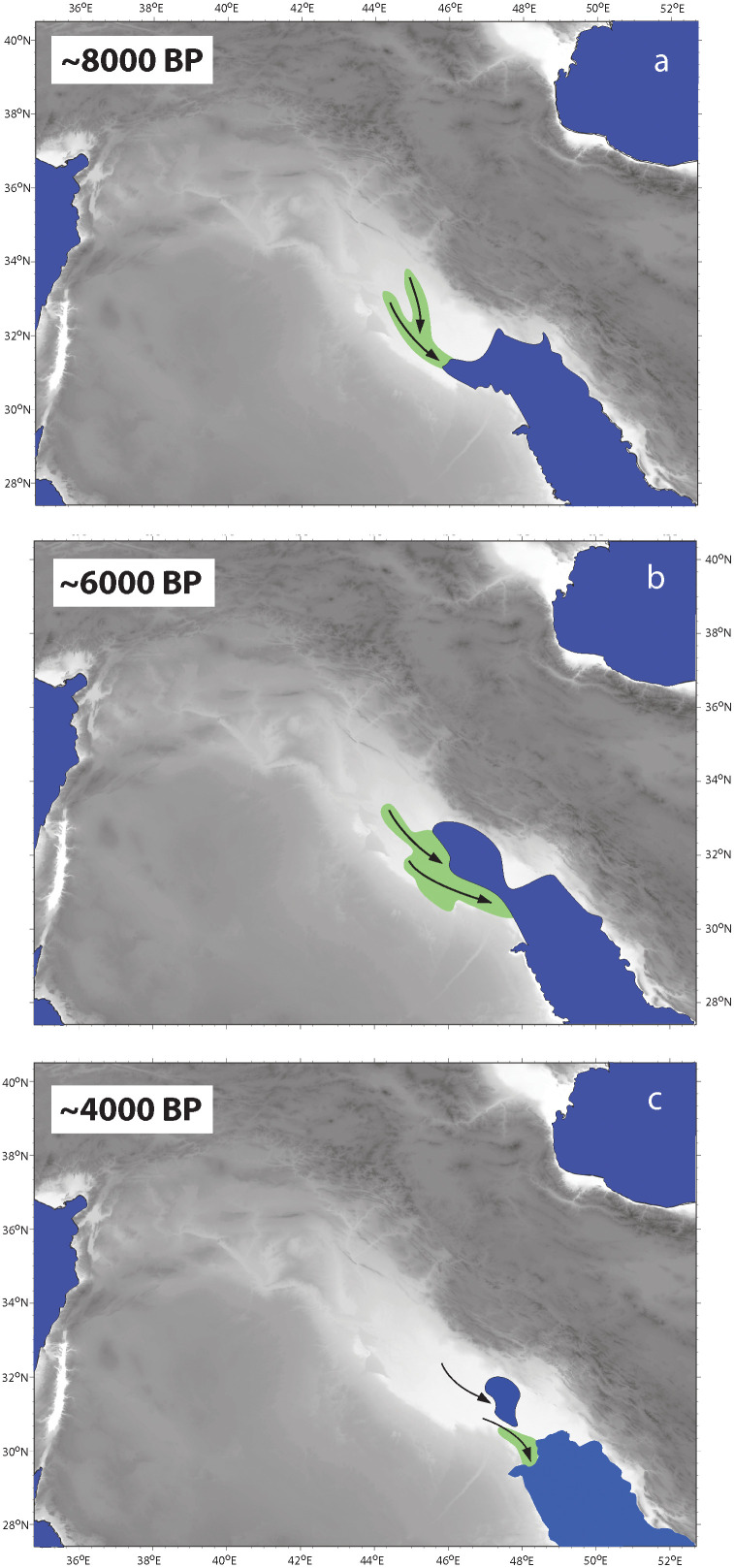
Sequential historical geography of coastal Mesopotamia at: a. 7,000, b. 6,000 and c. 4,000 years ago. Inferred directions of fluvial systems (black arrows) responsible for building the Mesopotamian delta complex are shown with their tidally-influenced zones (in green).

The maximum inland tidal reach must have occurred at the start of the highstand c. 7,000 years ago along the incised valleys of the Euphrates and Tigris (Fig 6a). However, the most laterally extensive tidal freshwater zone was probably established as the Sumer delta lobe developed its network of channels during highstand (Fig 6b), which corresponds to the later half of the Ubaid and early Uruk periods. The self-regulating irrigation and drainage system that the tidal pulse provides requires little human intervention and minimal periodic maintenance. Thus, the establishment of tidal irrigation with reduced subsistence risk could help explain the transition from the evenly dispersed settlements during the earlier Ubaid period to a proto-urban phase with structured hierarchies of sites by the middle Uruk [46,80]. Since no textual evidence exists for tides, their decline must have been substantial by late Uruk (c. 5,500 BP), which is consistent with our interpretation of geological data.

As access to the sea was restricted when the Euphrates lobe merged with the Khuzestan delta, the tidal freshwater zone moved downstream along the newly formed Shatt al-Arab channel(s) (Fig 6c). Tidal irrigation became thus impossible farther inland and the need to maintain economic and political power around established habitation centers probably led the adoption of large-scale fluvial irrigation. Similar to previously proposed scenarios, we suggest that favorable population concentration available to mobilize for intensive labor together with intensification of grain production “maximized the possibilities of appropriation, stratification, and inequality” [81] that ultimately led to the formation of the state structures. Rather than invoking a hypothetical aridity affecting fluvial discharge [82], we link this fundamental transition to environmental constraints imposed by large scale coastal morphodynamics. The stability provided by inherited tidal agricultural assets (e.g., fields, canals) as well as the cultural investments (i.e., temples and towns) of the proto-urban period was thus preserved through adopting and enhancing fluvial irrigation techniques.

## 5. Conclusions and perspectives

In addition to a longstanding tradition that explains ancient urbanization as a natural consequence of intensified subsistence systems where state-level social structures become innate [83], recent research draws attention to the importance of deltaic resources in the development of cities and states from the point of view of subsistence [41,84], trade [52], technology [85], demography [86], and sociopolitical institutions [87], most notably as they relate to hierarchy and economy, often based on theocratic and/or sociopolitical ideologies. As another key to the puzzle, we add here coastal morphodynamics as a fundamental control on early high-yield agricultural production – in turn supporting demographic growth with associated specialization – based on tidal irrigation. As tides declined, we argue that the evolving coastal landscape was a root cause – the environmental push – for adopting and enhancing large-scale river irrigation in coastal Sumer, and with that, contributing to the evolving character of Mesopotamian institutions and the emergence of what classically has been described as the State, all built around ensuring abundance [88].

By considering the rise and fall of tidal influence on coastal Sumer, some lines of inquiry open immediately. A first order problem is the control on tides exerted by the Khuzestani fan-delta and whether its transition from a fan morphology to a fast-growing delta was ultimately controlled by anthropogenically enhanced erosion in the Zagros Mountains. The location of Khuzestan Province, influenced early by highland domestication centers [89], upstream the delta, certainly justifies such a hypothesis given its long and continuous agricultural and pastoral history [e.g., 90].

Although tides are not textually remembered or are still hidden behind deciphering ambiguities of the proto-cuneiform texts, it is warranted to ask if their memory was preserved in the earliest Sumerian mythology. For example, the cosmogonical role of Enki, the Sumerian god of water, in separating “sweet” from “bitter” waters [91] suggests a link to the dual freshwater/saltwater character of tidal circulation. As the patron of Eridu, the earliest first mythological “antediluvian” city in coastal Sumer, Enki’s archetypal temple at Eridu offered a model for all Mesopotamian temples. Its association with Abzu, the primordial freshwater source/spring from the deep, may have originated as an explanation for the rise and fall of tides in the Eridu Depression rather than inspired by artesian springs, which are not occurring naturally in the area.

The traumatic event of a universal flood recounted in the Eridu Genesis [92], may have indeed been inspired by actual events triggered by the dramatic landscape metamorphosis of coastal Sumer. Rather than rememorating the transgression, a process too slow for generational time scales, or localized fluvial floods [93], we argue that restricting the access to the sea magnified the inundation from annual spring floods of Tigris and Euphrates. Similar to large historical floods [94], sluggish evacuation of the blocked Mesopotamian Bay [95] may have led to prolonged inundation of mythical proportions on large swaths of coastal Sumer when peak floods from both rivers overlapped.

Refinement of the links between the environmental context and Sumerian economy, society and culture is expected with future detailed paleogeographical and paleohydrologic reconstructions combined with modeling of tides as well as with considering morphodynamics in future interpretations of existing and newly deciphered Mesopotamian texts.

## Supporting information

S1 File Inclusivity in global research statement. (DOCX)

S1 Table Br and S content. (DOCX)

S2 Table Total organic carbon content. (DOCX)

S3 Table AMS ^14^C dates. (DOCX)
