# Supplementary material for: Morphodynamic Foundations of Sumer
Source: PLoS One. 2025 Aug 20;20(8):e0329084. doi: 10.1371/journal.pone.0329084 (PMC12367111; doi:10.1371/journal.pone.0329084)
Supplement: S1 Table — (DOCX) [file pone.0329084.s002.docx]

**S1 Table.** Br and S content (counts per second) from Itrax XRF scanning of Lagash drill core.

| **depth (m)** | **Br (cps)** | **S (cps)** |
| --- | --- | --- |
| 0.35 | 117 | 2751 |
| 0.47 | 199 | 1073 |
| 0.63 | 160 | 645 |
| 0.70 | 189 | 209 |
| 0.80 | 155 | 408 |
| 1.27 | 134 | 1171 |
| 1.79 | 66 | 390 |
| 2.26 | 112 | 306 |
| 2.75 | 126 | 243 |
| 3.34 | 127 | 298 |
| 3.76 | 174 | 388 |
| 4.00 | 181 | 351 |
| 4.70 | 153 | 193 |
| 5.33 | 148 | 346 |
| 5.96 | 163 | 200 |
| 6.62 | 215 | 141 |
| 7.21 | 215 | 171 |
| 7.75 | 206 | 600 |
| 8.23 | 149 | 303 |
| 8.75 | 135 | 555 |
| 9.32 | 194 | 248 |
| 9.88 | 116 | 159 |
| 10.46 | 181 | 220 |
| 11.02 | 187 | 277 |
| 11.50 | 169 | 242 |
| 11.98 | 195 | 393 |
| 12.19 | 209 | 201 |
| 12.30 | 208 | 138 |
| 13.15 | 104 | 379 |
| 14.25 | 204 | 474 |
| 14.75 | 139 | 207 |
| 16.75 | 194 | 234 |
| 17.25 | 137 | 264 |
| 17.60 | 69 | 429 |
| 18.51 | 198 | 570 |
| 19.75 | 187 | 224 |
| 19.97 | 173 | 212 |
| 21.05 | 194 | 976 |
| 21.51 | 144 | 300 |
| 21.66 | 103 | 1894 |
| 22.30 | 83 | 1959 |
| 22.50 | 74 | 3838 |
| 23.50 | 49 | 572 |
| 23.75 | 37 | 250 |
| 24.10 | 68 | 306 |
