# Supplementary material for: Morphodynamic Foundations of Sumer
Source: PLoS One. 2025 Aug 20;20(8):e0329084. doi: 10.1371/journal.pone.0329084 (PMC12367111; doi:10.1371/journal.pone.0329084)
Supplement: S2 Table — (DOCX) [file pone.0329084.s003.docx]

**S2 Table.** Total organic carbon content from Lagash drill core samples.

| **Depth (m)** | **TOC [%]** |
| --- | --- |
| 0.58 | 0.43 |
| 0.95 | 0.55 |
| 1.28 | 0.47 |
| 1.78 | 0.41 |
| 2.25 | 0.42 |
| 2.55 | 0.39 |
| 2.88 | 0.41 |
| 3.84 | 0.42 |
| 4.1 | 0.38 |
| 4.54 | 0.42 |
| 4.87 | 0.42 |
| 5.27 | 0.5 |
| 5.47 | 0.47 |
| 5.99 | 0.52 |
| 6.5 | 0.46 |
| 6.7 | 0.53 |
| 7.07 | 0.45 |
| 7.37 | 0.57 |
| 7.55 | 0.64 |
| 7.9 | 0.39 |
| 8.2 | 0.35 |
| 8.55 | 0.38 |
| 8.8 | 0.5 |
| 9.12 | 0.42 |
| 9.25 | 0.43 |
| 10.23 | 0.46 |
| 10.51 | 0.43 |
| 10.8 | 0.52 |
| 10.98 | 0.43 |
| 11.47 | 0.46 |
| 12.01 | 1.18 |
| 12.3 | 1.13 |
| 13.15 | 0.56 |
| 14.25 | 0.52 |
| 14.75 | 0.4 |
| 16.75 | 0.43 |
| 17.25 | 0.39 |
| 17.75 | 0.42 |
| 18.56 | 0.43 |
| 19.8 | 0.37 |
| 20.3 | 0.44 |
| 21 | 0.41 |
| 21.24 | 0.39 |
| 21.55 | 0.38 |
| 22.4 | 0.42 |
| 23.5 | 0.41 |
| 24.68 | 0.41 |
| 25 | 0.44 |
