# Supplementary material for: Morphodynamic Foundations of Sumer
Source: PLoS One. 2025 Aug 20;20(8):e0329084. doi: 10.1371/journal.pone.0329084 (PMC12367111; doi:10.1371/journal.pone.0329084)
Supplement: S3 Table — (DOCX) [file pone.0329084.s004.docx]

**S3 Table.** AMS ^14^C dates of organic materials from Lagash drill core.

| **Altitude (m asl)** | **Type** | **14C Age (years BP)** | **14C Age Error (years)** | **Terrestrial Calibration Age (years)*** | **Marine Calibration Age (years)*** | **Calibrated range (ky)** |
| --- | --- | --- | --- | --- | --- | --- |
| 4.2 | Freshwater Mollusk | 590 | 15 | 544 - 635 |  | 0.5 - 0.7 |
| -1.8 | *Cerithium* fragment | 6040 | 20 | 6,830 - 6,950 | 5,905 - 6,260 | 5.9 - 7.0 |
| -1.9 | *Cerithium sp.* | 6140 | 20 | 6,945 - 7,158 | 5,980 - 6,360 | 5.9 - 7.2 |
| -2.3 | *Cerithium* fragment | 5950 | 25 | 6,676 - 6,880 | 5,778 - 6,181 | 5.7 - 6.9 |
| -2.7 | carbonized plant fragment | 5780 | 25 | 6,498 - 6,654 |  | 6.4 - 6.7 |
| -5.7 | *Cerithium* fragment | 5980 | 25 | 6,742 - 6,888 | 5,818 - 6,211 | 5.8 - 6.9 |
| -7.3 | leaf fragment | 5950 | 25 | 6,675 - 6,854 |  | 6.6 - 6.9 |
| -7.3 | *Cerithium sp.* | 6090 | 25 | 6,856 - 6,872 | 5,932 - 6,296 | 5.9 - 6.9 |
| -11.8 | mollusk fragment | 6,270 | 25 | 7,156-7,265 | 6,135-6,514 | 6.1 - 7.2 |

*Calendar ages are relative to year 2016
